# Relationships of Ischemic Stroke Occurrence and Outcome with Gene Variants Encoding Enzymes of Tryptophan Metabolism

**DOI:** 10.3390/biomedicines9101441

**Published:** 2021-10-11

**Authors:** Fanni Annamária Boros, Rita Maszlag-Török, Mónika Szűcs, Ádám Annus, Péter Klivényi, László Vécsei

**Affiliations:** 1 Department of Neurology, Faculty of Medicine, Albert Szent-Györgyi Clinical Center, University of Szeged, H-6725 Szeged, Hungary; boros.fanni@med.u-szeged.hu (F.A.B.); torok.rita@med.u-szeged.hu (R.M.-T.); annus.adam@med.u-szeged.hu (Á.A.); klivenyi.peter@med.u-szeged.hu (P.K.); 2 Department of Medical Physics and Informatics, Faculty of Science and Informatics, Albert Szent-Györgyi Medical School, University of Szeged, H-6720 Szeged, Hungary; szucs.monika@med.u-szeged.hu; 3 MTA—SZTE Neuroscience Research Group, H-6725 Szeged, Hungary; 4 Interdisciplinary Excellence Center, University of Szeged, H-6725 Szeged, Hungary

**Keywords:** gene expression, genotyping, IDO1, ischemic stroke, KYAT1, kynurenine pathway, SNP, stroke etiology, Trp metabolism, TPH1

## Abstract

Ischemic stroke is among the leading causes of mortality and long-term disability worldwide. Among stroke risk factors the importance of genetic background is gaining interest. There is a growing body of evidence of changes of metabolite levels and enzyme activities involved in the conversion of Trp during the course of cerebral ischemia. We compared the frequencies of ten SNPs of five genes related to Trp metabolism between groups of 122 ischemic stroke patients and 120 control individuals. Furthermore, we examined the mRNA levels of *TPH1*, *IDO1* and *KYAT1* genes in peripheral venous blood with the aim of assessing (i) whether there are changes in their expression during the course of stroke and (ii) does any of their investigated SNPs have an impact on gene expression. In seven cases out of ten studied polymorphisms we detected significant differences in frequencies in relation to ischemic stroke occurrence, etiology, and clinical parameters. We also detected changes in the expression of *TPH1* and *IDO1* genes during the course of the disease. We found that those IDO1 variants which show a trend towards elevated mRNA level are more frequent in stroke patients than in controls. Our results are important novel observations which suggest a causal relationship between elevated *IDO1* expression and stroke etiology.

## 1. Introduction

Stroke is the fourth leading cause of death worldwide and is also among the leading causes of long-term adult disability [1]. The majority of stroke cases are ischemic, when the occlusion of a blood vessel leads to the damage of the corresponding brain tissue.

Despite the striking improvement of acute stroke management during past decades involving thrombolytic treatment, thrombectomy and stroke inpatient units, stroke related mortality and morbidity continue to be major health concerns [2]. The often very severe and detrimental consequences of stroke carry a great burden not only because of physical disability but also due to the resulting decline in cognitive and emotional abilities and arising healthcare costs [3]. Therefore, prediction of stroke outcome in an early stage is pivotal in order to implement early rehabilitation when necessary.

It is well accepted that ischemic stroke is a multifactorial disease which has both modifiable and non-modifiable risk factors. In the group of modifiable risk factors belong diabetes, hypertension, obesity, hyperlipidemia and smoking, among several others [1]. Among the non-modifiable risk factors, the importance of genetic background is gaining interest. There are various genetic disorders that include ischemic stroke as manifestation, such as Fabry disease (variants of alfa galactosidase A gene [4]); Marfan syndrome (*FBN1* gene) [5]; Sickle cell disease (*HBB* gene) [6]). In addition to these several genes have been identified so far of which have variants with a pathogenic role in stroke occurrence (reviewed in [1]). Examples of these are variants of *NOTCH3* [7] and *HTRA1* [8]. Next to the well-established causal mutations, there is a growing body of evidence of genetic factors that have, or are proposed to have a stroke risk modifying effect. Among potential risk factor gene variants are mutations of genes related to apoptosis, such as *FOXF2* (transcription factor forkhead box protein F2) [9], genes involved in immune responses and central nervous system (CNS) autoimmunity as *TSPAN2*, a member of the tetraspanin family [10], and regulators of gene expression, such as *HDAC9* (Histone Deacetylase 9), *PITX2* (paired-like homeodomain transcription factor 2) and *ZFHX3* (Zinc Finger Homeobox 3) (reviewed: [1]). Gene variants related to stroke not only affect the likeliness of the occurrence of an ischemic event but can also influence stroke outcome. Mutation of *CCR5* (Chemokine receptor 5, a pro-inflammatory G protein coupled receptor) was shown to have an impact on recovery after stroke, and was proposed as a potential therapeutic target after brain injury [11].

**Figure 1:**
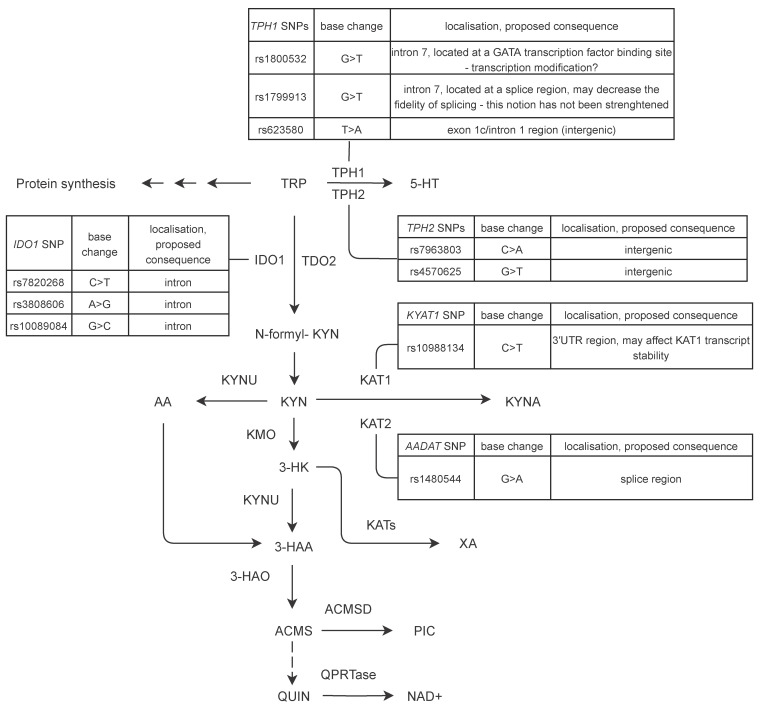
Schematic outline of Trp metabolism towards the production of serotonin and the kynurenine pathway. Enzymes and metabolites important in respect of this study are only shown by bold capital and capital abbreviations, respectively. The investigated variants of five genes included in this study are listed next to the corresponding gene. For references of SNP localisation and proposed consequences of the polymorphisms see Figure 2 and Figure 3 and text. Abbreviations: TPH1: tryptophan hydroxylase 1; TPH2: tryptophan hydroxylase 2; 5-HT: 5-hydroxytryptamine, serotonin; IDO1: indoleamine 2,3-dioxygenase 1; TDO2: tryptophan 2,3-dioxygenase; KYN: kynurenine; KYAT1: kynurenine aminotransferase 1 (gene); UTR: untranslated region; KAT1: kynurenine aminotransferase 1; KAT2: kynurenine aminotransferase 2; AADAT: kynurenine/alpha-aminoadipate aminotransferase; KYNU: kynureninase; AA: anthranilic acid; KYNA: kynurenic acid; KMO: kynurenine monooxygenase; 3-HK: 3-hydroxy-kynurenine; 3-HAA: 3-hydroxyanthranilic acid; XA: xanthurenic acid; 3-HAO: 3-hydroxyanthranilate 3,4-dioxygenase; ACMS: aminocarboxymuconate semialdehyde; ACMSD: aminocarboxymuconate semialdehyde decarboxylase; PIC: picolinic acid; QUIN: quinolinic acid; QPRTase: quinolate acid phosphoribosyltransferase; NAD+: nicotinamide adenine dinucleotide.

The kynurenine pathway (KP) is the main route of tryptophane (Trp) metabolism (Figure 1), in which among others indoleamine 2,3-dioxygenase 1 (IDO1), tryptophan 2,3-dioxygenase (TDO2), kynureninase (KYNU), kynurenine aminotransferases (KATs) and kynurenine monooxygenase (KMO) enzymes catalyze reactions by which various neuroactive metabolites are produced (reviewed in [12]). Among the metabolites kynurenic acid (KYNA) exerts neuroprotective effects via various mechanisms, while 3-hydroxy-kynurenine (3HK) and quinolinic acid (QA) are known for their neurotoxic properties (for a review see [13]).

Up-regulation of the pathway has been reported repeatedly in both preclinical and clinical studies of ischemic stroke (reviewed: [2]). Darlington and colleagues showed that increased Trp catabolism is initiated immediately after the ischemic event, which is likely due to the inflammatory response and oxidative stress. The most prominent change was manifested in 3-hydroxyanthranilic acid (3-HAA) level in strong correlation with infarct volume. It was proposed that oxidative Trp metabolism could be a contributor to post-stroke oxidative stress and brain damage [14]. In line with that, Brouns et al. reported up-regulation of the KP in the hyperacute phase of ischemic stroke. The activity of the KP was found to correlate with stroke severity and long-term outcome, as there was a correlation between the KYN/Trp (kynurenine/Trp) ratio at admission (which is an indicator of IDO1 activity) and National Institutes of Health Stoke Scale (NIHSS) score at admission and infarct volume. In the case of those patients who showed higher Trp metabolism on admission, a less favorable stroke outcome was observed. Furthermore, KP activity was also found to correlate with the level of inflammatory markers such as C-reactive protein (CRP) and erythrocyte sedimentation rate [15].

In a recent study Hajsl et al. reported alterations of various KP enzymes upon ischemic brain injury. They found that in patients suffering from ischemic stroke Trp, KYN and 3-HAA levels were lower, while 3HK and anthranilic acid (AA) levels were higher compared to control individuals. In accord with changes of metabolite levels, activities of KP enzymes differed as well: IDO1, KMO, 3-hydroxyanthranilate 3,4-dioxygenase (3-HAO) and the composed 3-HAO and aminocarboxymuconate semialdehyde decarboxylase (ACMSD) activity was higher in stroke patients compared to controls [16].

In addition to the unambiguous results indicating enhanced Trp metabolism, recently the KP attracted further interest in relation to stroke due to the realization of the importance of inflammatory processes in the pathomechanism of CNS disorders [2,17]. Mo and colleagues found that in addition to lower Trp, KYNA and KAT activity, CRP levels and IDO activity were significantly higher among stroke patients compared to control individuals [18]. IDO activity positively correlated with CRP, moreover, both CRP levels and IDO activity showed positive correlation with stroke severity.

The relationship between ischemic stroke and the KP is not restricted to enzyme activity and metabolite level changes: recently Wigner et al. investigated the distribution frequency of variants of 5 genes related to tryptophan metabolism in ischemic stroke patients and control individuals and found significant difference in the frequencies of seven out of the ten investigated variants [19].

In this study we aimed to determine whether (1) differences in the frequency of occurrence of selected variants of five Trp metabolism related genes can be found between stroke and control patients and in comparisons made between subgroups of stroke patients differing in regard of inflammatory markers, Trial of Org 10172 in Acute Stroke Treatment (TOAST) classification, short-term stroke outcome, (2) does the presence of a specific single nucleotide polymorphism (SNP) have an effect on stroke progression and inflammatory parameters, (3) which clinical parameter(s) if any from a selected group of them has/have an effect on short term stroke outcome (4) does any of the studied genetic variations have an effect on the expression of the affected gene before or after stroke treatment and (5) is there a specific pattern in the change of TPH1, IDO1 and/or KYAT1 mRNA levels during treatment of stroke patients.

## 2. Materials and Methods

### 2.1. Participants

The frequency of ten variants of five Trp metabolism related genes were investigated in groups of 122 ischemic stroke patients and 120 control individuals. Participants of the control group had no medical history of ischemic event of the CNS.

Demographic data of the study groups are summarized in Table 1.

All patients involved in this study were hospitalized in the Stroke Department of the Neurology Clinic, University of Szeged between January 2018 and November 2019, and underwent systemic thrombolytic therapy. Thrombectomy was also peformed in selected cases with large vessel occlusion. Detailed clinical data for the patients were obtained from the STAY ALIVE acute stroke registry, which is a prospectively collected, national, hospital-based multicenter database of acute ischemic stroke patients. Written informed consent was obtained from each patient or their proxy. Data regarding alcohol intake and smoking habits were collected from patients’ questionnaires. Blood samples drawn upon arrival at the Stroke Department were analyzed for renal function, lipid profile, and inflammatory markers in an accredited diagnostic laboratory of the clinic. The cutoff values of CRP and white blood cell (WBC) count were determined based on the reference range of the diagnostic laboratory. The diagnosis of diabetes (diabetes mellitus; DM) was based on fasting glycaemia ≥126 mg/dL (7 mmol/L) and/or current antidiabetic treatment. Hypertension (HT) was defined as blood pressure >140/90 mmHg and/or current antihypertensive treatment. CT and MRI scans were analyzed by experienced neuroradiologists. ECG analysis, TOAST classification, modified Rankin Scale (mRS) and NIHSS score evaluation were carried out by specialized vascular neurologists. Clinical data available for the stroke study group is summarized in Table 2.

For data analysis the group of stroke patients was divided into subgroups based on age, short term disease outcome (based on the difference of the NIHSS score at admission and discharge) and levels of inflammatory markers. For description of study groups and subgroups used for statistical comparisons please see Table 3.

Gene expression analysis was carried out from samples of 55 patients of the above described stroke study group (average age ± SD: 67.75 ± 9.38 years, male/female ratio: 34/21).

From each participant included in the study informed consent was obtained. The study protocol was approved by the Medical Research Council Scientific and Research Ethics Committee and was in full accordance with the Declaration of Helsinki ethical principles for medical research involving human subjects.

### 2.2. Sample Collection and Processing

For RNA and DNA analysis, 6 mL peripheral venous blood was obtained from stroke patients at three time points using ethylenediamine-tetraacetic acid (EDTA) containing tubes (BD Vacutainer K2E (EDTA)). Collection of the first sample set was immediately before systemic thrombolytic therapy (sample set ‘A’). Samples of the ‘acute sample set’ were collected 2 to 6 h after the intervention (sample set ‘B’). Samples of the third sample set (sample set ‘C’) were collected in the subacute phase, 10 to 15 h following thrombolysis.

### 2.3. DNA Isolation, Genotyping

For genotyping, genomic DNA was isolated from 500 μL of peripheral blood collected before thrombolytic therapy. In the case of three participants blood for DNA isolation was not available from sample set ‘A’, thus samples collected in either the acute or subacute phase were used. For genomic DNA extraction a simple desalting method was implemented (based on the protocol described in [20]). Extracted DNA was stored at −20 °C till further use. As for control samples, isolated DNA stored at the Biobank of our Institute were used. The method of extraction and storage conditions of control samples were identical to as described above.

For SNP analysis commercially available TaqMan allelic discrimination assays (Thermo Fisher Scientific Inc., Marietta, OH, USA) were used. Cycling conditions were the following: 95 °C for 10 min, 95 °C for 15 s and 60 °C for 1 min, the latter two steps repeated 40 times.

### 2.4. RNA Isolation, Gene Expression Analysis

For RNA isolation 6 mL venous blood was treated with lysis buffer (containing NH_4_Cl_6_, EDTA, KHCO_3_ and DEPC). Following repetitive cycles of washes with lysis buffer, white blood cells were collected by sedimentation and lysed in 1 mL Tri reagent (Molecular Research Center Inc., Cincinnati, OH, USA) with repetitive pipetting. Samples were then stored at −80 °C until further use. Total RNA isolation was carried out according to the protocol of the Tri reagent manufacturer. RNA concentration was determined using MaestroNano micro-volume spectrophotometer (MaestroGenInc, Hsinchu, Taiwan). Prior to cDNA synthesis, 500 ng RNA was treated with RNase-free DNase I (Thermo Fisher Scientific Inc., Marietta, OH, USA) for elimination of genomic DNA. cDNA was synthesized using random hexamer primers with Revert Aid First Strand cDNA Synthesis Kit according to the instructions of the manufacturer (Thermo Fisher Scientific Inc., Marietta, OH, USA). Quantitative PCR (qPCR) reactions were carried out using SYBER green detection (RT2 Syber Green Mastermix, qPCRBIO, PCR Biosystems Ltd., London, UK) in a final volume of 20 μL of which the cDNA template was 1 μL. For *IDO1* and *TPH1* expression analysis undiluted cDNA samples were used, for other reactions 10-fold diluted cDNA templates were used. For reference gene 18S rRNA level was determined. RT-qPCR (reverse transcription-qPCR) reactions were carried out in a CFX96 thermocycler (Bio-Rad). For primer sequences and cycling conditions please see Table 4.

### 2.5. Statistical Analysis

For the analysis of allele distributions and genotype frequencies PLINK software was used [21,22]. Chi-square or Fisher’s exact tests were implemented and odds ratio (OR) with 95% confidence interval (CI) were calculated. Similarly, for the investigation of the relation of clinical parameters and short term disease outcome either Chi-square tests or independent t tests were implemented.

For the analysis of haplotype blocks and haplotype-based case–control analysis the Haploview 4.2 software [23,24] was used.

Analysis of RT-qPCR results was performed using the ΔΔCt method. In brief, ΔCt was calculated as the difference between a gene of interest and the average of the reference gene. ΔΔCt was calculated as ΔCt (affected individual)—average ΔCt (reference individuals). Fold change was determined as 2^−ΔΔCt^ value [25].

For the statistical analysis of differences of 2^−ΔΔCt^ replicates of different study groups GraphPad Prism 6.01 statistics software was used [26]. For analysis of data distribution, the D’Agostino & Pearson omnibus normality test was implemented.

When analyzing gene expression during acute and subacute phases of stroke, samples obtained before systemic thrombolysis served as reference. Since the analysis consisted of repeated measurements and the data differed from Gaussian distribution, the nonparametric Friedman test was used.

When comparing gene expression changes in relation with different genotypes fold change of individuals homozygous for the wild allele served as reference, and the nonparametric Kruskal-Wallis test was implemented.

In both analysis setups Dunn’s test was used for multiple comparisons.

## 3. Results

### 3.1. Genotype and Allele Distribution of the Investigated SNPs

We determined the frequencies of a total of ten polymorphisms of five genes encoding key enzymes of Trp metabolism (Figure 1) in groups of ischemic stroke patients and matching healthy controls. None of the investigated SNPs showed significant deviation from Hardy Weinberg equilibrium (*p* < 0.01) and the minor allele frequencies (MAFs) of all variants were comparable to the MAFs available on public databases regarding European populations [27].

**Figure 2:**
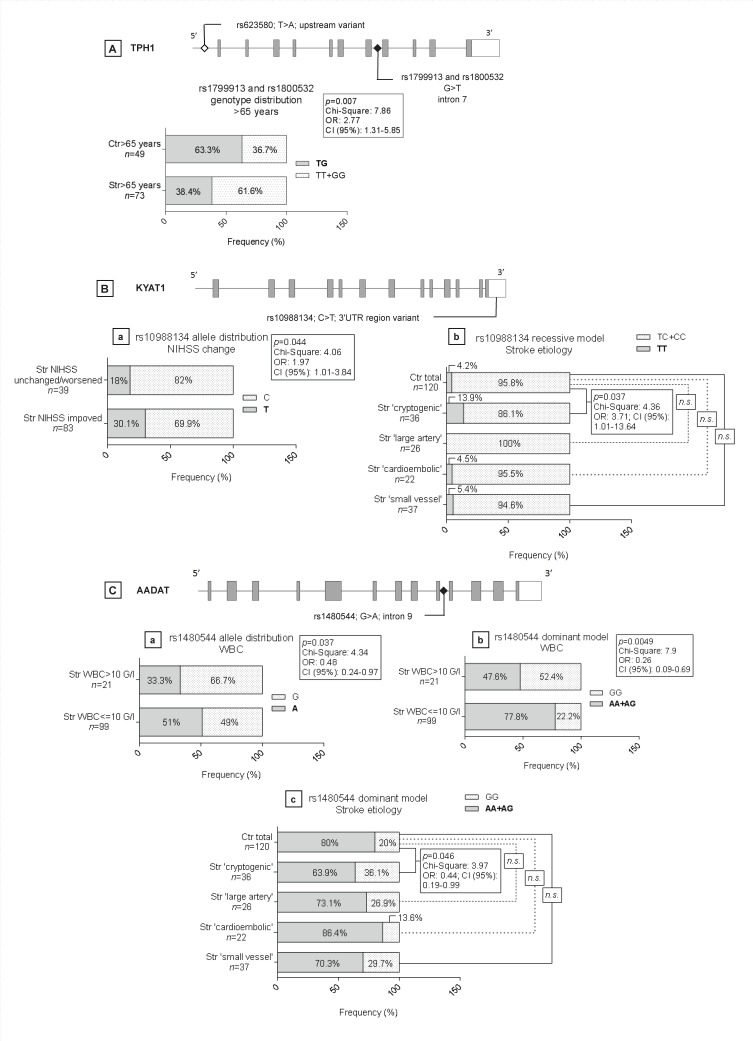
Detected significant differences in the frequencies of studied *TPH1* (**A**), *KYAT1* (**B**) and *AADAT* (**C**) gene variants. Gene structures are illustrated with grey boxes indicating exons and small rhombuses the positions of SNPs studied. Graphs of allele frequencies are shown only for comparisons of groups and subgroups of stroke patients and controls where significant differences were detected (**a**–**c**), as indicated by statistical data in boxes. For a detailed description please see the text. Abbreviations: TPH1: tryptophan hydroxylase 1; Ctr: control; Str: stroke; OR: odds ratio; CI: confidence interval; KYAT1: kynurenine aminotransferase 1; NIHSS: NIH Stroke Scale; UTR: untranslated region; AADAT: kynurenine/alpha-aminoadipate aminotransferase; WBC: white blood cell; n.s.: non-significant.

**Figure 3:**
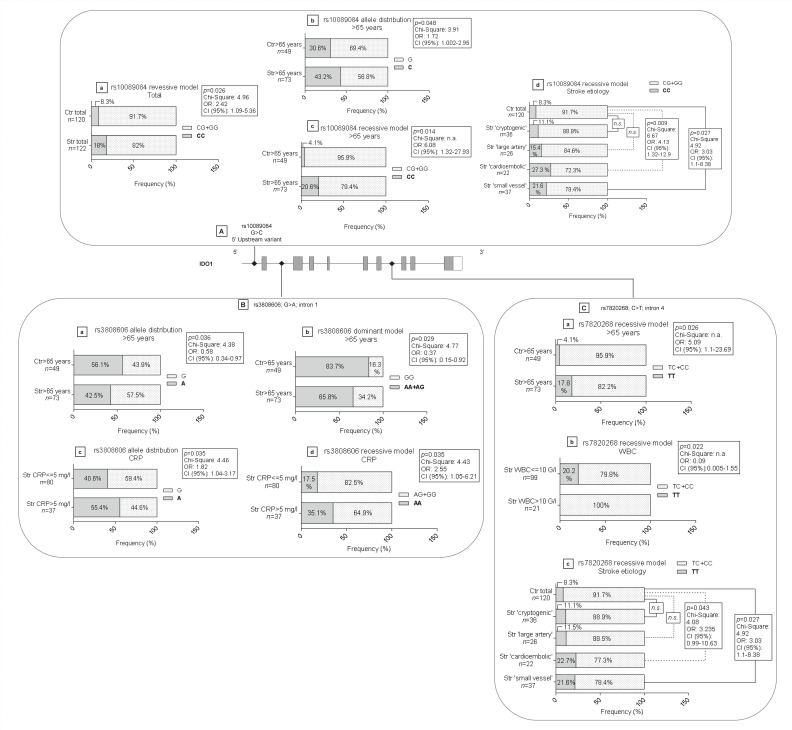
Detected significant differences in the frequencies of the studied *IDO1* variants (**A**–**C**). Gene structure is illustrated with grey boxes indicating exons and small rhombuses the positions of SNPs studied. Graphs of allele frequencies are shown only for comparisons of groups and subgroups of stroke patients and controls where significant differences were detected (**a**–**d**), as indicated by statistical data in boxes. For a detailed description please see the text. Abbreviations: IDO1: indoleamine 2,3-dioxygenase 1; Ctr: control; Str: stroke; OR: odds ratio; CI: confidence interval; CRP: C-reactive protein; WBC: white blood cell; n.s.: non-significant.

The positions and types of genetic alterations within the studied genes together with the expected effects of nucleotide changes are shown in Figure 1. Frequencies of the studied SNPs were compared between whole groups of healthy and stroke patients as whole sample groups and between subgroups containing subjects with age over 65. Similarly, subgroups of stroke patients were assembled for SNP frequency comparisons based on WBC, CRP levels, TOAST classification and short term disease outcome (NIHSS score change) (Table 3). Detected significant differences in the frequencies of the studied SNPs in different comparisons between various subgroups of stroke patients and controls are summarized in Figure 2 and Figure 3, and detailed below.

#### 3.1.1. *TPH1* Variants rs1799913, rs1800532 and rs623580

The frequencies of three *TPH1* SNPs were assessed. The SNPs rs1800532 and rs1799913 are localized in the 7th intron of the *TPH1* gene. They are proposed to be located at a GATA transcription binding site and a splice region thus are proposed affecting transcription and decreasing the fidelity of splicing, respectively [19]. Rs623580 is a variant upstream to the *TPH1* gene coding region.

We found rs1799913 and rs1800532 to be in linkage disequilibrium (LD), as they always occurred simultaneously among both stroke patients and control individuals. When comparing the genotype distribution of these *TPH1* variants between group Str > 65 years and the corresponding control group, a significant difference was detected in the case of both SNPs (*p* = 0.023) (Figure 2A). This is the result of a significantly higher ratio of the variant in heterozygous form among control individuals compared to the stroke cohort (TG vs. TT + GG; TG (Str > 65 years): 38.35% vs. TG (Ctr > 65 years): 63.26%; *p* = 0.007). No significant differences were detected in genotype or allele frequencies of these two SNPs in any other comparisons. The frequency of the rs623580 SNP of the *TPH1* gene did not show significant difference in any of the comparisons.

#### 3.1.2. *TPH2* Variants rs7963803 and rs4570625

The two investigated *TPH2* variants, rs7963803 and rs4570625 are located in the 5′-UTR and flanking region of gene. We did not detect significant difference in either the allele, or the genotype frequencies of these two investigated *TPH2* SNPs in any of the implemented comparisons (data not shown).

#### 3.1.3. *KAT1* Variant rs10988134

The rs10988134 variant of KAT1 (*KYAT1*) is located in the 3′-UTR region of the gene and has been proposed to affect the stability of the KAT1 transcript [28]. We found the SNP to be more frequent among stroke patients whom status improved during their stay at the clinic after stroke treatment (Str (NIHSS improved); T allele: 30.12%) compared to those patients who showed no improvement in their NIHSS score (Str (NIHSS unchanged/worsened); T allele: 17.95%) (*p* = 0.044) (Figure 2B(a)). The variant was also significantly more frequent in the recessive model (TT vs. TC + CC) among patients with cryptogenic stroke etiology compared to the control group (TT Control: 4.2% vs.TT Str ‘cryptogenic’: 13.9%; *p* = 0.037) (Figure 2B(b)). There was no significant difference in the distribution of either allele or genotype frequency in relation to this variant in comparisons made between any other subgroups.

#### 3.1.4. *AADAT* Variant rs1480544

The rs1480544 is an intronic variant of the *AADAT* gene (encoding KAT2). It is located in a putative exonic silencer region and is proposed to impact *AADAT* mRNA production [29]. The presence of the minor allele has been associated with decreased levels of various inflammatory markers in both blood and cerebrospinal fluid (CSF) [30].

We found that the minor allele (A) of the variant was significantly more frequent among stroke patients with normal WBC count (Str (WBC ≤ 10G/L)) compared to those with elevated WBC levels (Str (WBC >10G/L)) (51.01% vs. 33.33%, respectively; *p* = 0.037) (Figure 2C(a)). The genotype distribution of the variant also differed significantly when applying the same comparison (*p* = 0.017). This intergroup difference was the consequence of the minor allele of the variant being significantly more frequent in either homozygous or heterozygous form (AA + AG; dominant model) among Str (WBC ≤ 10 G/L) group (77.77%) compared to the Str (WBC > 10 G/L) subgroup (47.61%) (*p* = 0.0049) (Figure 2C(b)). The frequency of rs1480544 *AADAT* SNP also differed significantly applying the dominant model for comparison between patients with stroke of ‘cryptogenic’ etiology and control group (AA + AG Ctr: 80% vs. Str ‘cryptogenic’: 63.9%; *p* = 0.046) (Figure 2C(c)).

Regarding comparisons between other subgroups, no significant differences were detected in either allele or genotype distributions of the variant.

#### 3.1.5. *IDO1* Variants rs3808606, rs10089084 and rs7820268

We included three *IDO1* gene variants in our study. Each of these is located in intronic regions (Figure 3) and has been suggested by previous studies to occur with different frequencies in relation with stroke and/or have an impact on IDO1 mRNA levels [31].

The minor allele of rs10089084 *IDO1* SNP we found significantly more frequent in homozygous form (CC) in Str total group compared to Ctr total group (CC vs. CG + GG; Str (CC): 18% vs. Ctr (CC): 8.3 %; *p* = 0.026) (Figure 3A(a)). The minor allele of the variant was found also with a significantly higher frequency among patients (43.15% vs. 30.61%; *p* = 0.048) (Figure 3A(b)) in comparison of allele frequencies of stroke patients over 65 years (Str > 65 years) and corresponding controls (Ctr > 65 years). Regarding these two study groups there was also a significant difference in the genotype distribution of the SNP (*p* = 0.031). This was found to be the result of being the variant present in a significantly higher ratio in homozygous form among stroke patients compared to controls (CC vs. CG + GG; CC (Str >65 years): 20.55% vs. CC (Ctr >65 years): 4.1%; *p* = 0.014) (Figure 3A(c)).

The variant was also significantly more frequent in the recessive model among patients with TOAST classification of ‘small vessel stroke’ compared to control individuals (CC vs. CG + GG; CC Str (small vessel): 21.62% vs. Ctr total: 8.33%; *p* = 0.027). Between the groups of patients with stroke of cardioembolic etiology and non-stroke controls there was a significant difference in the genotype distribution of the variant (*p* = 0.029), which was the consequence of the SNP being present more frequently in the recessive model among stroke patients compared to controls (CC vs. CG + GG; CC Str (cardioembolic): 27.3% vs. Ctr total: 8.33%; *p* = 0.0098) (Figure 3A(d)).

We found that the major (G) allele of the rs3808606 SNP was more frequent in the group of stroke individuals over 65 years (Str > 65 years: 57.53%) compared to the corresponding control group (Ctr > 65 years: 43.88%) (*p* = 0.036) (Figure 3B(a)). Similarly, in comparison between the same groups in homozygous form the major allele (GG) was significantly more frequent among stroke patients (34.24%) compared to control individuals (16.32%) (GG vs. AA + AG; *p* = 0.029) (Figure 3B(b)).

Comparison between subgroups of stroke patients showed that the minor allele (A) of the rs3808606 variant was significantly more frequent among those patients whose CRP level exceeded 5 mg/L (Str (CRP > 5 mg/L) vs. Str (CRP ≤ 5 mg/L); 55.41% vs. 40.62%; *p* = 0.035) (Figure 3B(c)). Similarly, the minor allele in homozygous form (AA vs. AG + GG) was significantly more frequent among stroke patients with higher CRP level compared to those with CRP under 5 mg/L ((AA) Str (CRP > 5 mg/L) vs. (AA) Str (CRP ≤ 5 mg/L): 35.14% vs. 17.5%; *p* = 0.035) (Figure 3B(d)).

In case of *IDO1* SNP rs7820268 variant the minor allele was significantly more frequent in homozygous form (TT) in the Str >65 years subgroup compared to the Ctr >65 years subgroup (TT vs. TC + CC; (TT) Str > 65 years: 17.8% vs. (TT) Ctr > 65 years: 4.08%; *p* = 0.026) (Figure 3C/a). In addition, the genotype distribution of the variant differed between Str (WBC ≤ 10 G/L) and Str (WBC > 10 G/L) subgroups. This difference was a consequence of the variant being significantly more frequent in homozygous form (TT) in the group of stroke patients with WBC not exceeding 10 G/L (20.2%) compared to the Str (WBC > 10 G/L) subgroup, where the variant in homozygous form was absent (0%) (recessive model; *p* = 0.022) (Figure 3C(b)).

Similarly to as seen in the case of the rs10089084 *IDO1* variant, the rs7820268 SNP was also significantly more frequent in the recessive model (TT vs. TC + CC) among patients with TOAST classification of ‘small vessel stroke’ and ‘cardioembolic stroke’ compared to control individuals (TT (Str ‘small vessel’): 21.62% and TT (Str ‘cardioembolic’): 22.72% vs. TT (Ctr total): 8.33 %; *p* = 0.027 and 0.043, respectively) (Figure 3C(c)).

Apart from those mentioned above the frequencies of the investigated three *IDO1* variants did not show significant differences in any other comparison.

### 3.2. Haplotype Blocks

LD analysis revealed three haplotype blocks, one in *TPH1*, *TPH2* and *IDO1* genes each. Haplotype analysis of the identified LD blocks revealed no significant association with stroke and no significant difference was observed in case of any comparisons implemented between the subgroups shown in Table 3.

### 3.3. Relationship of Clinical Parameters and Short Term Stroke Outcome

In order to assess whether any of the clinical parameters has a predictive value for stroke outcome (based on changes in NIHSS scores) we investigated whether there is a significant difference in a set of clinical parameters among subgroups of stroke patients with improved NIHSS score and patients who showed no improvement or worsening of NIHSS score. The analyzed clinical parameters were the following: sex, age at disease onset, alcohol consumption, smoking, antiplatelet medication, DM, HgbA1c, door-to-needle (DtN) time, ECG (sinus rhythm or atrial fibrillation), TOAST classification, pre-stroke mRS score, HT, urea nitrogen (UN) and creatinine levels, low density lipoprotein (LDL), cholesterol, triglycerides, hyperlipidaemia.

The ratio of patients consuming alcohol among those who showed early improvement (based on NIHSS score change) was smaller compared to the group of stroke patients who showed no improvement/worsening in NIHSS score (26.2% versus 43.6%). Though this difference statistically showed marginal significance (*p* = 0.057; Chi square: 3.63; OR (95%CI): 2.17 (0.97–4.86)), the difference between the ratios was prominent.

Apart from alcohol consumption, there was no difference between the two stroke subgroups in regard of any of the investigated clinical parameters listed above.

### 3.4. Gene Expression Analysis

We aimed to determine whether any of the SNPs investigated in this study has an effect on the expression of the corresponding gene. Furthermore, we also wished to learn whether changes are detectable in the expression of *TPH1*, *TPH2*, *IDO1*, *KYAT1* and *AADAT* genes in peripheral blood samples (PBS) of ischemic stroke patients during the course of the disease. To this end we analyzed mRNA levels of the studied genes using RT-qPCR. In PBS we could not detect *TPH2* and *AADAT* expression. This is in accord with data available on www.proteinatlas.org (accessed on 30 March 2021) as *TPH2* and *AADAT* show low or no expression in human blood cells. TPH1, IDO1 and KYAT1 mRNAs on the other hand were detectable in our samples, thus we compared levels of these normalized to 18S rRNA as a reference which showed small variation throughout all samples (Ct standard deviation: ±0.72).

#### 3.4.1. Effects of Gene Variants on mRNA Levels in Blood Samples

None of the investigated *TPH1* (rs1800532, rs1799913 and rs623580) or *KYAT1* (rs10988134) variants showed an effect on the expression of the corresponding gene (Supplementary Materials Figures S1 and S2).

**Figure 4:**
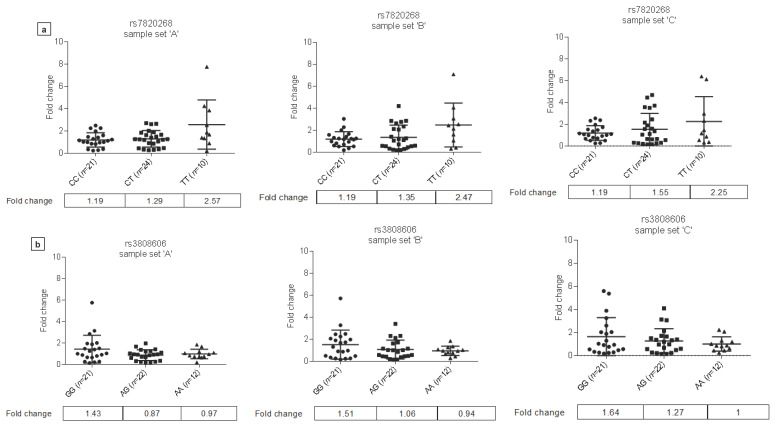
Effects of *IDO1* variants on IDO1 mRNA level in peripheral blood samples of ischemic stroke patients. *IDO1* rs7820268 (T) variant (**a**) and rs10089084 (C) variant (not shown) result in a trend towards elevated IDO1 mRNA level in peripheral blood samples of stroke patients, while IDO1 rs3808606 A variant (**b**) is associated with a tendency of reduced mRNA level. Blood samples were drawn before thrombolysis (sample set A) and 2–6 h, and 10–15 h after thrombolysis (sample sets B and C, respectively). The variants of *IDO1* form a haplotype block, and rs7820268 and rs10089084 show strong linkage disequilibrium. Since the latter two variants result in similar changes in mRNA level, only data for rs7820268 SNP are presented.

*IDO1* SNPs rs7820268 and rs10089084 resulted in a clearly observable trend towards increased IDO1 mRNA level in the presence of either of the two variants, though the difference was not significant (rs7820268 fold changes: ‘A’ sample set: CC: 1.19, CT: 1.29, TT: 2.57; ‘B’ sample set: CC: 1.19, CT: 1.35, TT: 2.47; ‘C’ sample set CC: 1.19, CT: 1.55, TT: 2.25; rs10089084 fold changes: ‘A’ sample set: GG: 1.21, CG: 1.33, CC: 2.37; ‘B’ sample set: GG: 1.19, CG: 1.44, CC: 2.32; ‘C’ sample set: GG:1.21, CG:1.59, CC: 2.14) (Figure 4a).

On the contrary, the presence of *IDO1* rs3808606 SNP showed a tendency of decreasing mRNA level: those bearing the minor A allele of the variant showed decreased gene expression compared to those who were of homozygous wild genotype. The difference was more prominent when comparing IDO1 expression between homozygotes for the minor and major allele of rs3808606 polymorphism (fold changes: ‘A’ sample set: GG: 1.43, AA: 0.97; ‘B’ sample set: GG: 1.51, AA: 0.94; ‘C’ sample set: GG: 1.64, AA: 1.0) (Figure 4b).

#### 3.4.2. Changes in TPH1, IDO1 and KYAT1 mRNA Levels during the Course of Ischemic Stroke

**Figure 5:**
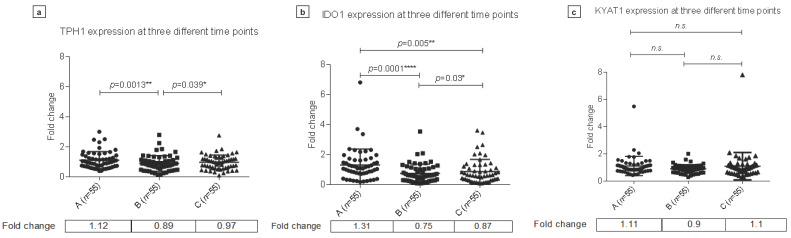
Changes in TPH1, IDO1 and KYAT1 mRNA levels during the course of ischemic stroke. Changes in TPH1 (**a**), IDO1 (**b**) and KYAT1 (**c**) mRNA levels in peripheral blood samples of ischemic stroke patients drawn before thrombolysis (A), in acut phase (B) and >10 h following thrombolysis (C). TPH1 expression is significantly lower in the acute phase following thrombolysis (B) compared to both before (A) and >10 h following treatment (C). IDO1 is significantly up-regulated in samples collected prior to thrombolytic therapy (A) both in comparison with samples obtained in the acute (B) and subacute phase (C) following intervention. No significant difference is detectable in KYAT1 mRNA levels by comparing samples in different time points during the course of the disease; *p* < 0.05; * *p* < 0.01; ** *p* < 0.001; **** *p* < 0.0001.

Comparisons of mRNA levels at three time points of ischemic stroke revealed that *TPH1* expression was significantly lower in the acute phase following thrombolysis (fold change ‘B’: 0.89) compared to both before (fold change ‘A’: 1.12) and following >10 h (fold change ‘C’: 0.97) the treatment (*p* = 0.0013 and 0.039, respectively) (Figure 5a).

*IDO1* was significantly up-regulated in samples collected prior to thrombolytic therapy (fold change: 1.31) both in comparison with samples obtained in the acute (fold change: 0.75) and subacute phases (fold change: 0.87) following intervention (A vs. B *p* = 0.0001 and A vs. C *p* = 0.005) (Figure 5b). Interestingly, the lowest level *IDO1* expression was detected in samples collected in the acute phase after thrombolytic treatment, similarly to as seen in the case of *TPH1* expression change. Gene expression at this time point compared to fold changes in samples drawn >10 h after intervention showed significant difference (fold regulation 0.75 vs. 0.87, *p* = 0.03) (Figure 5b).

No significant differences were detectable in the expression of *KYAT1* by comparing samples of different time points in the course of the disease (Figure 5c).

## 4. Discussion

KP is the main route of Trp metabolism along which several neuroactive metabolites are produced. Several of these possess neuroprotective or neurotoxic properties and metabolite levels of the pathway as well as alterations in activities of the participating enzymes have been reported in various neurological disorders [17]. Interconnection among changes in Trp metabolism and ischemic stroke has also been reported repeatedly from preclinical and clinical studies indicating accelerated Trp metabolism in relation with the disease [13,32]. However, data regarding the role and effects of genetic alterations of Trp metabolism related genes in ischemic stroke are scarce.

In this study we compared the frequencies of ten SNPs of five genes related to Trp metabolism between groups of ischemic stroke patients who underwent systemic thrombolytic therapy/thrombectomy and control individuals. Furthermore, we examined if any of the investigated SNP affected the mRNA level of the corresponding gene in peripheral venous blood and also compared the mRNA levels of three of the analyzed genes (*TPH1*, *IDO1* and *KYAT1*) in blood samples of stroke patients collected at three different time points during the course of the disease.

In seven cases out of ten studied polymorphisms we detected significant differences in frequencies in relation to ischemic stroke occurrence, disease etiology, inflammatory markers and short-term disease outcome.

Tryptophan hydroxylase (TPH1) is a protein of 444 amino acids which catalyzes the transformation of Trp into 5-hydroxytryptophan, a precursor to serotonin synthesis. The *TPH1* gene is located on the short arm of chromosome 11, consisting of 11 exons. Two SNPs of *TPH1* included in our study (rs1799913 and rs1800532) are variations within intron 7, localized in close proximity to each other. It is assumed, that these variants affect the transcription or splicing of TPH1 mRNA [19]. Up to date the effect of these SNPs have been studied in relation with personality traits [33,34], addiction [35,36] and schizophrenia [37]. We found rs1799913 and rs1800532 SNPs in complete LD. This observation is in accord with results from other study populations [38]. Our findings of the variants being more frequent in heterozygous form in the control group compared to the group of ischemic stroke patients is also in line with findings of Wigner et al. [19].

Expression changes of *TPH1* in relation with stroke have been investigated in animal models of the disease. In the in vivo MCAO (middle cerebral artery occlusion) rat model of ischemic stroke, *TPH1* was up-regulated in brain tissue of the animals 4 days after artery occlusion [39]. As far as we are aware, studies focusing on changes of *TPH1* expression in stroke patients have not been conducted so far.

TPH1 plays a central role in the peripheral production of serotonin—a monoamine, which has a crucial role in homeostasis via regulating vasoconstriction and platelet function [40]. TPH1 deficient mice were found to lack peripheral serotonin synthesis, thus showing diminished thrombosis and thromboembolism risk [40]. This gave ground to attempts of gene therapy targeting *TPH1* down-regulation with the aim of treating thrombotic disorders. Peter and colleagues developed a TPH1 specific miniribozyme, by which selective TPH1 mRNA cleavage was achieved resulting in decreased biosynthesis of serotonin [41]. These findings suggest that the observed increase in *TPH1* expression preceding thrombolytic therapy might not be a consequence, but rather a risk factor of occurring thrombosis.

The *IDO1* gene is located on the short arm of chromosome 8 spanning 14,900 nucleotides. It encodes the 403 amino acid IDO1 protein, which catalyses the first and rate limiting step of Trp metabolism via the KP by transforming the amino acid into N-formyl-L-kynurenine. IDO1 plays a central role in the production of immuno- and neuroactive KP metabolites, underlining its importance in immunomodulation and inflammatory processes affecting the CNS.

*IDO1* rs3808606 SNP is a G to A change in intron 1 of the gene [42]. The impact of the variant on gene expression was studied in human bronchial epithelial cells of cystic fibrosis patients [31]. Lower mRNA levels were reported in cells bearing the TT/AA genotype compared to those with CC/GG genotype. Furthermore, defective enzyme activity and elevated levels of the pro-inflammatory citokine IL-6 were detected in patients with TT genotype. These data are in line with our results as we detected a trend towards decreased *IDO1* gene expression in the presence of the rs3808606 A allele. Furthermore, we found the A allele (both in allele frequency and in homozygous genotype) to be more frequent among stroke patients who had CRP levels exceeding the cutoff 5 mg/L. This observation is in line with findings of elevated IL-6 levels of patients homozygous for the minor allele [31], since IL-6 affects CRP levels by inducing gene expression and release of the protein both from liver and immune cells [43,44,45].

*IDO1* rs10089084 (G to C) SNP is an intronic variant located near the 5′ end of the gene. We found *IDO1* rs10089084 minor allele to be associated with higher mRNA level. To our knowledge this is the first study to investigate the effect of this gene variant on the expression of IDO1 mRNA.

The minor allele of the variant was reported to be significantly more frequent in homozygous form in healthy controls compared to ischemic stroke patients in a study of Polish population [19]. On the contrary, our data suggest the SNP to have a stroke risk increasing effect, since we found the minor allele of the variant in homozygous form to be more frequent among stroke patients. The SNP was also present at higher frequencies among patients with stroke of cardioembolic etiology or stroke as a consequence of small vessel occlusion when compared to the control group. However, no such association was detected in the case of large-artery atherosclerosis or cryptogenic etiology. These results underline the importance of taking into account the etiology of ischemic stroke during interpretation of data of genetic analyses regarding effects on the disease. This notion is further strengthened by our finding of marginal association of the rs7820268 *IDO1* variant (discussed below) with small vessel occlusion and cardioembolic etiology stroke, but not with other subtypes of the disease.

*IDO1* rs7820268 is a C to T change in intron 5 of the gene. The variant has been investigated in relation with multiple sclerosis [46], systemic sclerosis [47] and type 1 diabetes [42], however, to our knowledge this is the first study to assess the occurrence of the variant in relation with ischemic stroke.

Tardito and colleagues [47] reported an association between the minor allele (T) of the variant and impaired function of CD8+ T reg cells among systemic sclerosis patients. Our finding of a marginal association between the SNP and lower WBC count also suggests a link between this genetic variant and immune functions.

Recently the minor allele of the polymorphism has been reported to influence IDO1 mRNA expression. In line with our findings, Han et al. [48] detected up-regulated *IDO1* expression in the presence of the T allele in healthy human blood, putamen and occipital cortex samples. Interestingly, the effect of the variant on gene expression was found to depend on brain region, since the presence of the minor allele resulted in down-regulated *IDO1* expression in cerebellar cortex, substantia nigra, thalamus and hippocampus samples [48].

There is a growing body of evidence of ischemic event of the CNS resulting in immune alteration in the periphery leading to immune suppression [49,50]. However, the exact mechanisms by which the cross-talk between the CNS and peripheral organs arise are not fully understood yet [49]. A proposed possible mechanism of systemic immune response after acute ischemic injury of the CNS is that such injuries induce rapid systemic stress response activation leading to the generation of a systemic acute phase response [51], which involves release of pro-inflammatory cytokines and activation of immune cells [52].

IDO1 well could be playing a role in these processes as IDO1 up-regulation can be observed in various inflammatory states and IDO1 is induced by pro-inflammatory cytokines, such as IFNɣ, TNFα and IL-1β [53,54]. Besides inflammation, up-regulation of *IDO1* expression was found to be induced by stress, resulting in Trp and serotonin depletion in the plasma of stressed mice [55]. Stress related IDO1 up-regulation is dependent on the pro-inflammatory response induced by the stressor, since by anti-TNFα and IFNɣ treatment *IDO1* gene expression could be blocked [55]. These findings are in line with our results of increased IDO1 mRNA levels preceding systemic thrombolytic therapy (an early phase of ischemic stroke).

Enhanced IDO1 activity/expression leads to a more immune tolerogenic state [54] due to Trp depletion and also via converting mature dendritic cells into tolerogenic antigen-presenting cells [56]. This gives ground to the notion that IDO1 inhibitor therapy after the ischemic event could be beneficial against stroke-induced immunodepression and could help to decrease the occurrence of the so often fatal post-stroke infections.

KATs catalyze the generation of the neuroprotective KYNA, and their altered activity has been reported in various neurological disorders. Modulation of KAT activity is an appealing therapeutic approach for restoring the balance of KP metabolites [57]. In human, four KAT isoforms are expressed differently in different tissues.

KAT1 (glutamine transaminase K/cysteine conjugate beta-lyase 1) is a 422 amino acid protein encoded by *KYAT1* gene on the long arm of chromosome 9.

The rs10988134 SNP (C/T) is a 3′UTR variant of the gene which is proposed to affect the stability of KAT1 transcript [19]. The minor allele of the polymorphism in homozygous form (AA alias TT) was reported to increase the occurrence of depression in the Polish population [58]. The frequency of the SNP was assessed in a further study involving Polish stroke patients as well, however, no association was revealed between the occurrence of the variant and the disease [19].

Our findings are in line with those of Wigner and colleagues, since we found rs10988134 with similar frequencies in the groups of stroke patients and control individuals. Within the stroke group however, the variant was significantly more frequent among patients who showed early improvement compared to those who showed no improvement based on NIHSS score changes. To the best of our knowledge this is the first study showing differing occurrence of the variant in regard of short term ischemic stroke outcome.

KAT2 (aminoadipate aminotransferase) enzyme is encoded by *AADAT* gene, localized on the long arm of chromosome 4. KAT2 is a 425 amino acid protein which shows low or no expression in peripheral blood cells (The human protein atlas, www.proteinatlas.org, accessed on 30 March 2021), however it plays a principal role in the formation of KYNA in the CNS [59]. The variant has been investigated in relation with ischemic stroke [19], infectious diseases such as HIV and bacterial meningitis [30,60], and depression [58].

Wigner et al. [19] reported the variant to be associated with ischemic stroke, however, in our study we did not detect significant differences in the frequency of the variant between various study groups.

The effect of *AADAT* rs1480544 SNP on immune response and on inflammatory marker levels was investigated in patients with bacterial meningitis. The minor allele of the variant was reported to be more frequent in homozygous form among bacterial meningitis patients as compared to healthy individuals. Furthermore, the presence of the minor allele was associated with decreased cell count and TNFα, IL-1β, IL-6, MIP-1α/CCL3 and MIP-1β /CCL4 levels in CSF samples of patients. These data suggest that *AADAT* rs1480544 variant leads to impaired leukocyte recruitment in bacterial meningitis patients [30].

Interestingly, we also observed the minor allele of the rs1480544 variant to be more frequent among stroke patients with lower (<10 G/L) WBC count compared to those whose WBC count exceeded 10 G/L.

Our findings of both the investigated KAT1 and KAT2 variants occurring with different frequencies when comparing stroke patients with cryptogenic etiology compared to the group of control individuals strengthen the notion of the importance of patients’ stratification based on disease etiology in the evaluation of effects of gene variations.

A limitation of this study is the relatively small sample size, especially when comparing stroke patients’ subgroups of disease etiology. Thus further studies involving larger number of participants with well defined disease etiology are required. Furthermore, it still remains a question, if there is a direct relationship between altered mRNA levels and the disease, and/or with which subgroups of stroke does such a linkage exist. In order to clarify these questions, research focusing on elucidating the effects of these SNPs not only on gene expression but also on protein levels and enzymatic functions are highly warranted.

## 5. Conclusions

We present data demonstrating associations between ischemic stroke and variants of four genes (*TPH1*, *IDO1*, *KYAT1* and *AADAT*) encoding enzymes of Trp metabolism. Most significantly, we proposed direct causal relationship between IDO1 function and stroke, by showing that *IDO1* variants showing a trend towards elevated mRNA level are more frequent in stroke patients than in controls, while on the contrary, *IDO1* variants resulting in a trend towards decreased mRNA level are present among stroke patients less frequently than in non-stroke controls. Noteworthy is that association of these *IDO1* variants with stroke is recognizable primarily in groups of patients with advanced age, altered WBC and CRP levels and with particular stroke etiology, all of which could be directly related with altered IDO1 functions. These are important novel observations as thought elevated *IDO1* expression in stroke has been described earlier our report is the first suggesting causal relationship between IDO1 and stroke etiology.

## 6. Key Summary Points

- Despite the striking improvement of acute stroke management ischemic stroke is still among the leading causes of mortality and long-term disability worldwide.
- Our aim was to investigate the possible association between ischemic stroke and alterations of Trp metabolism-related genes in regard to both genomic variants and gene expression changes.
- Out of the ten studied polymorphisms we detected significant differences in the frequencies of seven variants in relation to ischemic stroke occurrence, disease etiology, inflammatory markers, and short-term disease outcome.
- We detected changes in TPH1 and IDO1 mRNA levels during the course of the disease.
- IDO1 variants showing a trend towards elevated mRNA level were more frequent in stroke patients than in controls, while IDO1 variants which lead to a trend of decreasing mRNA level are present among stroke patients less frequently than in non-stroke controls.
- Our results are important novel observations which suggest a causal relationship between elevated *IDO1* expression and stroke etiology.

## Figures and Tables

**Table 1:** Demographic data of members of the genotyping study groups.

Group	*n*	Age (Mean ± SD; Years)	Male/Female
Control (Ctr)	120	64.94 ± 9.46	57/63
Ctr > 65 years	49	74.31 ± 6.75	16/33
Stroke (Str)	122	67.22 ± 10.28	69/53
Str > 65 years	73	74.19 ± 4.7	37/36
Str TOAST: Large-artery atherosclerosis	26	68.46 ± 9.45	20/6
Str TOAST: Small-vessel occlusion	37	65.95 ± 8.87	18/19
Str TOAST: Cardioembolic	22	72.68 ± 8.9	8/14
Str TOAST: Cryptogenic	36	64.47 ± 11.9	22/14
Str (CRP ≤ 5 mg/L)	80	67.54 ± 10.47	44/36
Str (CRP > 5 mg/L)	37	66.97 ± 10.45	22/15
Str (WBC ≤ 10 G/L)	99	68.21 ± 9.64	52/47
Str (WBC > 10 G/L)	21	61.95 ± 12.39	16/5
Str (NIHSS: improved)	83	67.8 ± 10.71	46/37
Str (NIHSS: unchanged/worsened)	39	65.82 ± 9.42	23/16

Abbreviations: SD: standard deviation; Ctr: control; Str: stroke; TOAST: Trial of Org 10172 in Acute Stroke Treatment; CRP: C-reactive protein; WBC: white blood cell; NIHSS: NIH Stroke Scale.

**Table 2:** Categorical (Table 2/**A**) and continuous (Table 2/**B**) clinical parameters of ischemic stroke patients involved in the study.

**(A)**				
**Clinical Parameter**		***n***	**%**	**Missing Data**
Alcohol consumption	No	81	68.07	3
	Yes	38	31.93	
Antiplatelet therapy	No	82	67.21	0
	Yes	40	32.79	
DM	No	86	70.49	0
	Yes	36	29.51	
ECG	SR	92	79.31	6
	AF	24	20.69	
TOAST classification	Cryptogenic	36	29.75	1
	Cardioembolic	22	18.18	
	Large-artery atherosclerosis	26	21.49	
	Small-vessel occlusion	37	30.58	
HT	No	16	13.11	0
	Yes	106	86.89	
CRP	≤5 mg/L	80	68.38	5
	>5 mg/L	37	31.62	
WBC	≤10 G/L	99	82.5	2
	>10 G/L	21	17.5	
HL	No	21	18.1	6
	Yes	95	81.9	
Previous stroke	No	97	79.51	0
	Yes	25	20.49	
Smoking	No	78	66.1	4
	Yes	40	33.9	
Thrombectomy	No	103	84.43	0
	Yes	19	15.57	
Baseline imaging	CT angio	64	52.46	0
	Noncontrast CT	32	26.23	
	MRI	26	21.31	
Type of lesion	Nonlacunar	60	72.29	39
**(B)**				
**Clinical parameter**		**average ± SD**		**missing data**
DtN (min)		60.13 ± 41.14		2
pre- stroke mRS (score)		0.95 ± 1		5
UN (mmol/L)		6.43 ± 2		2
Creat (μmol/L)		89.3 ± 23.86		2
WBC (G/L)		8.38 ± 2.34		2
LDL (mmol/L)		2.85 ± 0.94		15
Cholesterol (mmol/L)		4.78 ± 1.19		4
HgbA1c (%)		6.47 ± 1.4		26
TG (mmol/L)		1.56 ± 1.21		4
NIHSS baseline		8.05 ± 4.69		0
NIHSS discharge		5.79 ± 5.96		0

Abbreviations: DM: diabetes mellitus; SR: sinus rhythm; AF: atrial fibrillation; TOAST: Trial of Org 10172 in Acute Stroke Treatment; HT: hypertension; CRP: C-reactive protein; WBC: white blood cell; HL: hyperlipidaemia; SD: standard deviation; DtN: Door-to-Needle; mRS: modified Rankin Scale; UN: urea nitrogen; Creat: creatinine; WBC: white blood cell; LDL: low density lipoprotein; HgbA1c: haemoglobin A1c; TG: triglyceride; NIHSS: NIH Stroke Scale.

**Table 3:** Study groups and subgroups used for statistical comparisons of genotyping data.

**Group**	***n***	**versus**	**Group**	***n***
Str total	122		Ctr total	120
Str ≤ 65 years	49		Ctr ≤ 65 years	71
Str > 65 years	73		Ctr > 65 years	49
Str TOAST: Large-artery atherosclerosis	26		Ctr total	120
Str TOAST: Small-vessel occlusion	37		Ctr total	120
Str TOAST: Cardioembolic	22		Ctr total	120
Str TOAST: Cryprogenic	36		Ctr total	120
Str (CRP ≤ 5 mg/L)	80		Str (CRP > 5 mg/L)	37
Str (WBC ≤ 10 G/L)	99		Str (WBC > 10 G/L)	21
Str (NIHSS: improved)	83		Str (NIHSS: unchanged/worsened)	39

Abbreviations: Str: stroke; Ctr: control; TOAST: Trial of Org 10172 in Acute Stroke Treatment; CRP: C-reactive protein; WBC: white blood cell; NIHSS: NIH Stroke Scale.

**Table 4:** Primer sequences and cycling conditions implemented in gene expression analysis.

Primer Sequences		
**TPH1**	FW	5′-TGCAGACCATCCTGGCTTCA-3′
	REV	5′-GGAATACGGTTCCCCAGGTC-3′
**IDO1**	FW	5′-TCACAGACCACAAGTCACAG-3′
	REV	5′-GCAAGACCTTACGGACATCT-3′
**KYAT1**	FW	5′-GCCATCCCTGTCTCCATCTT-3′
	REV	5′-AGCGTGGCTTCATCCTTCAC-3′
**18S rRNA**	FW	5′-GCTTAATTTGACTCAACACGGGA-3′
	REV	5′-AGCTATCAATCTGTCAATCCTGTC-3′
**Cycling conditions**		
**TPH1**		
95 °C	3 min	
95 °C	15 s	40×
69 °C	30 s	
**IDO1**		
95 °C	3 min	
95 °C	15 s	40×
68.4 °C	30 s	
**KYAT1**		
95 °C	3 min	
95 °C	15 s	40×
64.8 °C	15 s	
**18S rRNA**		
50 °C	2 min	
95 °C	2 min	
95 °C	30 s	35×
57 °C	45 s	
72 °C	30 s	

Abbreviations: TPH1: tryptophan hydroxylase 1; IDO1: indoleamine 2,3-dioxygenase 1; KYAT1: kynurenine aminotransferase 1; 18S rRNA: 18S ribosomal RNA.

## Data Availability

Supporting data are available upon request.

## Supplementary Materials

The following are available online at https://www.mdpi.com/article/10.3390/biomedicines9101441/s1, Figure S1: Effects of the investigated TPH1 variants on TPH1 gene expression in peripheral blood samples of ischemic stroke patients, Figure S2: Effect of rs10988134 SNP on KYAT1 gene expression in peripheral blood samples of ischemic stroke patients.

## Abbreviations

18S rRNA	18S ribosomal RNA
3-HAA	3-hydroxyanthranilic acid
3-HAO	3-hydroxyanthranilate 3,4-dioxygenase
3HK	3-hydroxy-kynurenine
AA	anthranilic acid
ACMSD	aminocarboxymuconate semialdehyde decarboxylase
AADAT	kynurenine/alpha-aminoadipate aminotransferase
CI	confidence interval
CNS	central nervous system
CRP	C-reactive protein
CSF	cerebrospinal fluid
DM	diabetes mellitus
DtN	door-to-needle (time)
HT	hypertension
HL	hyperlipidaemia
IDO1	indoleamine 2,3-dioxygenase 1
KAT	kynurenine aminotransferase
KMO	kynurenine monooxygenase
KYAT1	kynurenine aminotransferase 1
KYN	kynurenine
KYNA	kynurenic acid
KYNU	kynureninase
KP	kynurenine pathway
LD	linkage disequilibrium
LDL	low density lipoprotein
mRS	modified Rankin Scale
NIHSS	National Institutes of Health Stroke Scale
OR	odds ratio
PBS	peripheral blood sample
RT-qPCR	reverse transcription-qPCR
QA	quinolinic acid
qPCR	quantitative polymerase chain reaction
SNP	single nucleotide polymorphism
TDO2	tryptophan 2,3-dioxygenase
TOAST	Trial of Org 10172 in Acute Stroke Treatment
TPH1	tryptophan hydroxylase 1
TPH2	tryptophan hydroxylase 2
UN	urea nitrogen
WBC	white blood cell
