# Supplementary material for: Relationships of Ischemic Stroke Occurrence and Outcome with Gene Variants Encoding Enzymes of Tryptophan Metabolism
Source: Biomedicines. 2021 Oct 11;9(10):1441. doi: 10.3390/biomedicines9101441 (PMC8533114; doi:10.3390/biomedicines9101441)
Supplement: Supplementary file 1 [file biomedicines-09-01441-s001.zip › biomedicines-1376043-supplementary.pdf]

biomedicines

Relationships of Ischemic Stroke Occurrence and Outcome with Gene Variants Encoding Enzymes of Tryptophan Metabolism

Supplementary Material

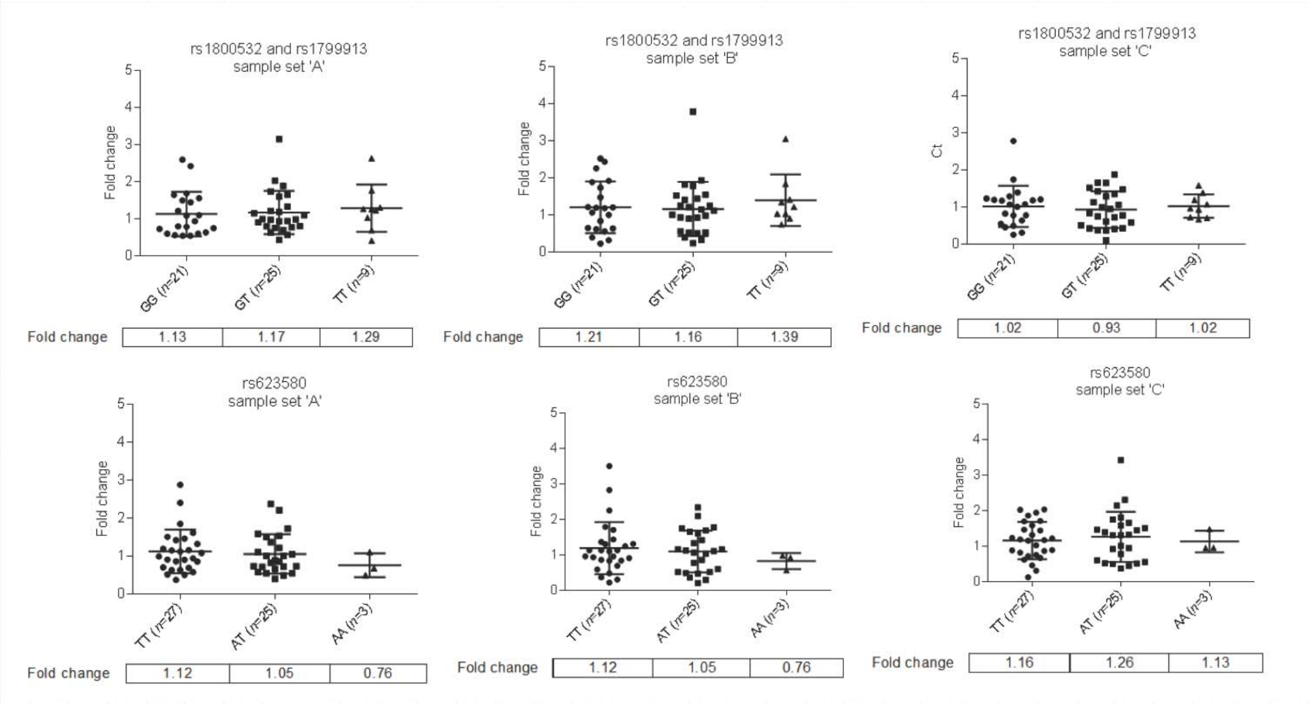

Figure S1. Effects of the investigated *TPH1* variants on *TPH1* gene expression in peripheral blood samples of ischemic stroke patients. None of the investigated TPH1 variants (rs1800532, rs1799913 and rs623580) showed an effect on the expression of the *TPH1* gene in peripheral blood samples of ischemic stroke patients.

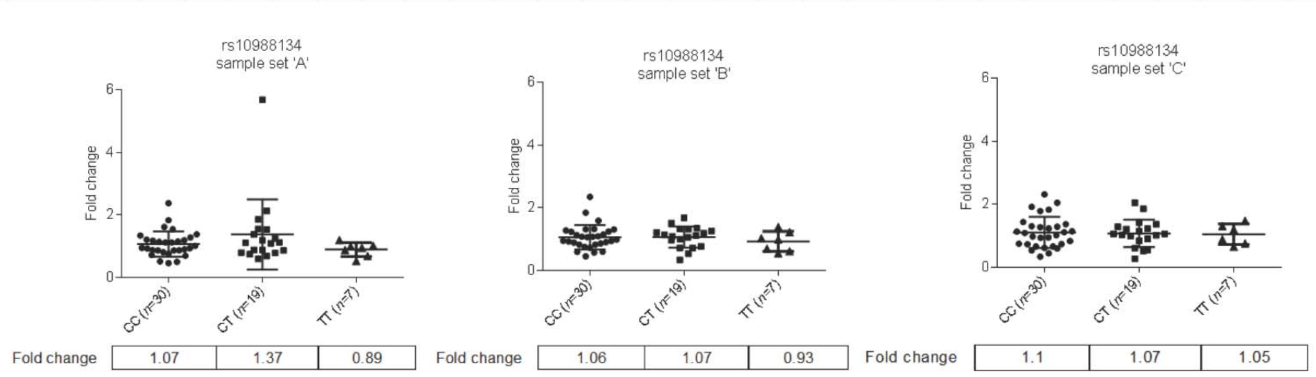

Figure S2. Effect of rs10988134 SNP on KYAT1 gene expression in peripheral blood samples of ischemic stroke patients. The investigated *KYAT1* rs10988134 variant did not show an effect on *KYAT1* gene expression in peripheral blood samples of ischemic stroke patients.
